# Challenges and Opportunities in Developing Standardized Dental Implant Curriculum for Nigeria's Dental Schools

**DOI:** 10.1002/jdd.13957

**Published:** 2025-07-17

**Authors:** Uvoh Onoriobe, Berna Saglik, Cortino Sukotjo, Marianella Sierraalta, Michael Razzoog, Sompop Bencharit

**Affiliations:** ^1^ Central Texas Veteran Healthcare System Temple Texas USA; ^2^ Biologic and Materials Sciences and Prosthodontics University of Michigan School of Dentistry Ann Arbor Michigan USA; ^3^ Department of Prosthodontics School of Dental Medicine University of Pittsburgh Pittsburgh Pennsylvania USA; ^4^ Workman School of Dental Medicine High Point University High Point North Carolina USA

**Keywords:** dental implants, implant dentistry, implant education, Nigeria, predoctoral education

## Abstract

**Introduction:**

Dental implant education in many African countries, including Nigeria, is limited due to a lack of expertise and resources. This study assessed the status of implant education in Nigeria's 13 dental schools, evaluated faculty and student willingness to implement a standardized curriculum, and explored student perceptions of implant education.

**Materials and Methods:**

A cross‐sectional study with two surveys was conducted in 2020. Survey I targeted faculty overseeing predoctoral curricula, while Survey II assessed dental students' perceptions of implant education. Primary outcomes included implant course offerings, faculty willingness to adopt an integrated curriculum, and student perceptions of implant training across fifth‐ and sixth‐year students and residents.

**Results:**

The response rate was 64% for Survey I and 65.6% for Survey II. Less than half (45%) of dental schools required implant education, though all faculty (100%) supported online implant courses. Barriers included financial constraints (27%), curricular time (36%), and lack of faculty expertise (27%). Implant courses were primarily taught by restorative departments (53%), with 88% delivered in the final (sixth) year. While 79.8% of students reported exposure to implantology, only 19% felt the coverage was sufficient. Interest in incorporating implants into future practice was high (92%, 87%, and 91% in fifth‐year students, sixth‐year students, and residents, respectively). However, formal implant education was lower among fifth‐year students (49%) compared to residents (81%, *p* < 0.001).

**Conclusions:**

There is a strong consensus to improve implant education in Nigeria. More comprehensive implant courses are needed to support future practitioners and enhance patient care.

## 1 Introduction

Recent decades of technological advancements in implant dentistry have rapidly expanded the field [1, 2, 3, 4]. In the United States, dental implant education was included in only 33% of dental schools in 1974 but had increased to 97% by 2004 [5, 6]. The McGill consensus, established over two decades ago, recognized implant overdentures as the standard of care for mandibular edentulism [7]. However, in Nigeria, implant dentistry remains limited in practice [8, 9]. The country, the most populous in Africa with over 230 million people, has 13 dental schools that produce approximately 185 dentists annually. As of 2000, there were 2598 licensed dentists in Nigeria, most of whom had limited training in dental implantology [10].

There is increasing awareness of dental implants as a treatment option among both the general population and dental providers in Nigeria [11]. Developing competent dentists capable of appropriate diagnosis, treatment planning, and implant therapy requires a comprehensive dental implant curriculum that integrates didactic instruction, simulation‐based training, and patient‐centered clinical experience [12, 13, 14, 15, 16]. However, 90% of dental schools in Nigeria have identified insufficient resources and lack of sponsorship as significant barriers to training both students and faculty in implant dentistry [15]. The importance of implant education is directly linked to its clinical application by graduate dentists [17, 18, 19]. Without comprehensive training aligned with the standard of care [20], there will be an insufficient number of competent and confident dentists to provide necessary implant therapy for Nigeria's large population. Furthermore, most graduate dentists in Nigeria have expressed dissatisfaction with the depth, knowledge, and training they received in implant dentistry [21].

Although the need to improve the dental implant curriculum and address the lack of training in Nigerian dental schools is widely recognized, the extent of these challenges, as well as faculty and student perceptions regarding curriculum improvements, remain insufficiently documented. This information is crucial for developing a standardized implant dentistry curriculum and implementing it effectively across all 13 dental schools in Nigeria. This study aims to assess the current status of predoctoral implant dentistry curricula in Nigeria through two objectives: (1) to evaluate the existing curriculum via faculty interviews and (2) to examine dental learners' perceptions of their implant education in relation to their training and future practice during the final 2 years of dental school and residency. The study hypothesizes that there is a statistically significant difference between the level of training and students' perceptions of implant education, as well as a statistically significant difference between the level of training and their attitudes toward implant practice.

## 2 Materials and Methods

This descriptive cross‐sectional study examined the educational details of implant courses offered through faculty interviews (Aim 1) and learners' attitudes and perceptions of the adequacy of implantology education in dental schools and their future implant practices (Aim 2). The first aim sought to establish a baseline understanding of the educational modalities and comprehensiveness of implant dentistry content from the faculty's perspective. The second aim evaluated whether the level of education (fifth and sixth dental students and residency as a predictor variable) predicted students' attitudes toward implant practices, implant education, and their expectations regarding dental implant prescription and practices.

The study protocol was modified from previous studies [13, 14, 15] that described the experiences in developing an integrated predoctoral implant curriculum in a large dental school, New York University College of Dentistry (NYUCD) and dental schools in Nigeria. The prior study [15] employed a basic survey distributed via email to 10 dental schools in Nigeria. In contrast, this study followed a similar survey approach but incorporated a faculty focus group, drawing methodology from two additional studies [13, 14]. The focus group was instrumental in refining and modifying the survey to align with the objectives of this more comprehensive study. By integrating faculty input, the survey was enhanced to capture a broader and more in‐depth perspective from both faculty members and learners, ensuring a more thorough and population‐specific assessment of implant education in Nigerian dental schools. The surveys, data collection, and analysis protocols were approved by Human Research Protection Program at the University of Michigan at Ann Arbor, IRB No. HUM00183694. The IRB approval was obtained specifically for the faculty and student surveys conducted in this study. The surveys were an initial planning part of the overall approach that consisted of four main stages: planning, implementation, program management, and post‐implementation. The consensus‐building pattern was utilized to bring together representatives from all Nigerian dental schools and to assess whether all parties agree to the general concept of a unified dental implant curriculum. A focus group with some leaders in dental education in Nigeria dental schools was also conducted via Zoom to discuss the importance and willingness of including implant dentistry in their curriculum. The focus group comprised two faculty members from each dental school who held leadership positions responsible for implant dentistry curricula, such as department chairs, course directors, or both. Participants were selected based on recommendations from their school's dean.

Several important questions were answered including the following:

1. Do you require the predoctoral students to take an implant dentistry course?
2. If a predoctoral implant course is not offered, why?
3. Which department offers the implant dentistry course?
4. Are any of the lectures available on the internet for the students to review?
5. Will you be willing to participate in an online course for your students?
6. Is it feasible for all schools to adopt a consensus dental implant curriculum?
7. Are you willing to participate in a training‐the‐trainer implant course?

After the focus group meeting, a survey consisting of 17 questions was administered via email to the key faculty members (*n* = 55) responsible for the predoctoral curriculum of all 13 dental schools in Nigeria. Two follow‐up/reminder emails were sent and thirty‐eight responses were received. The survey also included onset year, lecture hours, lectures available on the internet, required textbooks, department jurisdictions, the year of dental school the course was offered, clinical and laboratory courses, and implant systems used surgically and in the restorative phase.

A separate survey was administered via WhatsApp to students from 12 dental schools across the six geopolitical zones in Nigeria. The survey inquired about the need to have implant dentistry included in their curriculum and their perception of dental implant therapy. Three follow‐up/reminder messages were sent out. Eligible participants included all students in their last 2 years of training (Years 5 and 6) and residents‐in‐training. A total of 368 participants responded to the sent survey to 561 students/residents, representing a response rate of 65.6% across all universities. Of these, 37.8% and 46.7% were in their fifth and sixth year, respectively, and 15.5% were residents‐in‐training. For the student survey, participant identities were retained only until data collection was complete to prevent redundancy or duplicate entries. Afterward, all identifiable student information was permanently destroyed to ensure privacy. The data were not shared with dental schools or faculty.

Convenience sampling was employed for this study. For the faculty survey, the questionnaire was sent to the dean of each dental school, who identified the faculty member responsible for implant dentistry education. For the student survey, the questionnaire was distributed to the class with the assistance of the designated faculty member. Participation in the survey were voluntary. The analysis plan included descriptive and bivariate statistical methods to evaluate the current state of implant education in Nigerian dental schools. Descriptive statistics, including frequencies and percentages, were used to summarize the implant education curriculum across dental schools, faculty perspectives on integrating a standardized implant curriculum, and the existing implant education experiences of fifth‐ and sixth‐year dental students and residents. In addition, descriptive analyses assessed the perceived need for enhanced implant education, such as online learning modules. For the student survey, categorical variables, including student attitudes toward implant education, perceived adequacy of training, and expectations for incorporating implants into future practice, were analyzed using the chi‐square test. The chi‐square test was used to determine whether there were statistically significant differences in students' perceptions and attitudes based on their year of study (fifth‐ vs. sixth‐year students). This analysis assessed whether students in later years of training had different views on the adequacy of implant education and their readiness to practice implantology compared to those in earlier years. Bivariate analyses further examined associations between students' year of education and their willingness to pursue additional implant training, as well as their confidence in providing implant treatment postgraduation. Statistical significance was set at *p* < 0.05 for all tests. Data analyses were conducted using appropriate statistical software to ensure accuracy and reliability of results.

## 3 Results

### 3.1 Faculty Survey

A faculty survey was administered to faculty members in charge of the predoctoral implant curriculum of all 13 dental schools in Nigeria (Table 1). Eligible participants included all faculty from periodontics, oral maxillofacial surgery, and restorative dentistry who give implantology lectures in all 13 dental schools in Nigeria. The participants had diverse educational backgrounds, specializations, and years of experience. Thirty‐eight participants responded to the survey (of 55), representing a response rate of 69%. The survey had the highest number of respondents from the University of Benin (31.6%), followed by the University of Lagos (18.4%), Lagos State University (13.2%), and smaller numbers from various other universities across Nigeria. One of the 13 schools did not respond. A dental implant course was integrated into the required dental school education in more than half (55%) of all dental schools. The dental schools that reported no required implant curriculum, cited the lack of curriculum time to why implant courses (36%) or lack of financial resources and qualified faculty (27%). About half of dental schools incorporated implant dentistry in their curriculum between 2015 and 2017. A total of 33% commenced the implant curriculum prior to 2010. The dental implant course was given in the sixth year of dental curriculum in most schools (88%). Majority of implant education was taught by restorative faculty (53%) followed by oral and maxillofacial surgery faculty (27%), and periodontology faculty (20%). During the time of the survey, only 25% utilized internet‐based resources to augment the lectures given. All survey participants (100%) were interested in an additional online dental implant course for their students.

**TABLE 1:** Dental implant curricula based on faculty survey.

Do you require the predoctoral students to take an implant dentistry course?	
No	55%
Yes	45%
If a predoctoral implant course is not offered, why?	
Lack of financial resources	27%
Lectures incorporated in restorative course	9%
Lack of curriculum time	36%
Lack of qualified faculty	27%
Emphasis on postdoc	0%
Should not be in undergrad curriculum	0%
Long‐term concerns	0%
No clinical students	9%
If you do offer the implant course to the predoctoral students, when was it included in the curriculum?	
Prior to 2010	33%
2011–2013	0%
2015–2017	50%
2018 to present	17%
Which department offers the implant dentistry course?	
Department of restorative dentistry	53%
Department of periodontics	20%
Department of oral and maxillofacial surgery	27%
Others (please indicate)	
In what year is the course offered?	
Third year	
Fourth year	
Fifth year	
Sixth year	88%
Other	13%
What is the duration of the course?	
<2 months	63%
3–6 months	25%
7–12 months	0%
Nil	13%
Number of lectures	
<5	27%
6–10	27%
11–20	9%
21–30	0%
>30	9%
Are any of the lectures available on the Internet for the students to review?	
No	75%
Yes	25%
Will you be willing to participate in an online course for your students?	
Maybe	
Yes	100%
Are there required textbook(s) for the implant course?	
No	90%
Yes	10%

### 3.2 Learners' Survey Characteristics

The survey was distributed to all 13 schools of dentistry across the six geopolitical zones in Nigeria. Eligible participants included all students in their last 2 years of training (Years 5 and 6) and residents‐in‐training. A total of 368 participants responded to the survey (of 561), representing a response rate of 65.6% across all universities. Of these, 37.8% and 46.7% were in their fifth and sixth year, respectively, and 15.5% were residents‐in‐training. A new school did not have students and thus did not complete the student/resident survey. The time taken to complete the survey was modest with a mean of 6.9 min, a standard deviation of 30.8 min, and median of 3 min. In addition, most students responded to all items on the survey. The percentage of respondents with missing data was highest for questions related to implant treatments (range: 0.5%–1.6%) and lowest for questions pertaining to implant education (range: 0%–1.1%) and future plans related to dental practice (range: 0.2%–0.5%). Table 2 illustrates learner's perceptions on the prescription of implant therapy, while Table 3 demonstrates learner's perception on implant education and their future incorporation of implant dentistry into their practice.

**TABLE 2:** Learner's perception on implant therapy.

Characteristics	Total (*n* = 368)	Fifth year (*n* = 139)	Sixth year (*n* = 172)	Resident in training (*n* = 57)	*p* value
If your right mandibular first molar were missing, how would you like to have it restored? *n* (%)					0.25
Three‐unit fixed partial denture (bridge)	55 (15.2)	22 (16.1)	27 (16.1)	6 (10.5)	
With a removable partial denture	54 (14.9)	23 (16.8)	27 (16.1)	4 (7.0)	
With a single implant restoration	253 (69.9)	92 (67.2)	114 (67.9)	47 (82.5)	
If your right maxillary central incisor were missing, how would you like to have it restored? *n* (%)					0.02
With a 3‐unit bridge	31 (8.5)	10 (7.3)	18 (10.7)	3 (5.3)	
With a removable partial denture	59 (16.3)	29 (21.2)	28 (16.6)	2 (3.5)	
With a single implant restoration	273 (75.2)	98 (71.5)	123 (72.8)	52 (91.2)	
If your patient were edentulous in the mandible, how would you like to have it restored? *n* (%)					0.03
With a conventional complete denture	126 (34.4)	53 (38.4)	62 (36.3)	11 (19.3)	
With a fixed implant supported prostheses	158 (43.2)	57 (41.3)	76 (44.4)	25 (43.9)	
With an implant supported overdenture	82 (22.4)	28 (20.3)	33 (19.3)	21 (36.8)	

*Note: p* values are from a chi‐square test for categorical variables only.

**TABLE 3:** Learner's perception to implant education and future practice.

Characteristics	Total (*n* = 368)	Fifth year (*n* = 139)	Sixth year (*n* = 172)	Resident in training (*n* = 57)	*p* value
How did you first hear about implant treatment? (select all that apply)					
Internet, *n* (%)	119 (32.3)	55 (39.6)	49 (28.5)	15 (26.3)	0.07
Continuing education (CE) course, *n* (%)	38 (10.3)	9 (6.5)	12 (7.0)	17 (29.8)	< 0.001
School lecture, *n* (%)	237 (64.4)	68 (48.9)	123 (71.5)	46 (80.7)	< 0.001
Clinical instructor, *n* (%)	69 (18.8)	17 (12.2)	37 (21.5)	15 (26.3)	0.03
Classmate, *n* (%)	37 (10.1)	23 (16.5)	12 (7.0)	2 (3.5)	0.11
Other, *n* (%)	37 (10.1)	23 (16.5)	12 (7.0)	2 (3.5)	< 0.001
Has implant topics ever mentioned during your predoctoral dental education? *n* (%)					0.06
Yes	293 (79.8)	102 (73.4)	144 (83.7)	47 (83.9)	
How do you rate your level of knowledge about implant treatment? *n* (%)					0.001
Intermediate	139 (37.8)	40 (28.8)	68 (39.5)	31 (54.4)	
Limited	208 (56.5)	94 (67.6)	94 (54.7)	20 (35.1)	
Proficient	21 (5.7)	5 (3.6)	10 (5.8)	6 (10.5)	
Do you think implant topic is sufficiently covered during your undergraduate education? *n* (%)					0.307
Yes	71 (19.5)	22 (16.1)	43 (25.1)	6 (10.5)	
Do you think implant education should be taught at the undergraduate level? *n* (%)					0.72
Neither agree nor disagree	6 (1.6)	3 (2.2)	1 (0.6)	2 (3.5)	
Somewhat agree	68 (18.5)	27 (19.4)	33 (19.2)	8 (14.0)	
Somewhat disagree	1 (0.3)	0 (0.0)	1 (0.6)	0 (0.0)	
Strongly agree	290 (78.8)	108 (77.7)	135 (78.5)	47 (82.5)	
Strongly disagree	3 (0.8)	1 (0.7)	2 (1.2)	0 (0.0)	
If you agree, to what level should it be incorporated? *n* (%)					< 0.001
Theoretical	11 (3.1)	2 (1.5)	9 (5.4)	0 (0.0)	
Theoretical + simulated practical	33 (9.2)	12 (8.9)	12 (7.1)	9 (16.4)	
Theoretical + simulated practical + clinical observation of case	205 (57.3)	54 (40.0)	110 (65.5)	41 (74.5)	
Theoretical + simulated practical + clinical observation of case + clinical requirements	109 (30.4)	67 (49.6)	37 (22.0)	5 (9.1)	
Are you interested in learning more about implant treatment? *n* (%)					0.7
Maybe	30 (8.2)	13 (9.4)	14 (8.1)	3 (5.3)	
No	3 (0.8)	2 (1.4)	1 (0.6)	0 (0.0)	
Yes	335 (91.0)	124 (89.2)	157 (91.3)	54 (94.7)	
Which implant aspect interests you the most? *n* (%)					0.19
Both	231 (63.5)	93 (67.9)	96 (56.5)	42 (73.7)	
Neither	3 (0.8)	1 (0.7)	1 (0.6)	1 (1.8)	
Prosthetic	62 (17.0)	20 (14.6)	36 (21.2)	6 (10.5)	
Surgical	68 (18.7)	23 (16.8)	37 (21.8)	8 (14.0)	
How are you going to pursue your implant education?					
Continuing education course, *n* (%)	105 (28.8)	35 (25.5)	47 (27.5)	23 (40.4)	0.1
Specialty program, *n* (%)	255 (69.9)	98 (71.5)	120 (70.2)	37 (64.9)	0.65
From another dentist, *n* (%)	80 (21.9)	25 (18.2)	36 (21.1)	19 (33.3)	0.06
From dental implant company, *n* (%)	53 (14.5)	19 (13.9)	16 (9.4)	18 (31.6)	0.002
Other, *n* (%)	36 (11.4)	21 (15.8)	19 (11.0)	7 (12.3)	
Are you planning to incorporate your future practice with implant treatment? *n* (%)					0.28
Yes	327 (89.3)	126 (92.0)	149 (86.6)	52 (91.2)	
Would you be interested in an online course on dental implant treatment? *n* (%)					0.74
Yes	343 (93.5)	131 (94.2)	158 (92.4)	54 (94.7)	

*Note: p* values are from a chi‐square test for categorical variables only.

### 3.3 Learner's Perceptions on Implant Therapy

Most trainees believe missing mandibular first molar and maxillary central incisors should be restored with a single implant restoration; 82.5% and 91.2% for the residents‐in‐training, 67.2% and 71.5% for fifth‐year dental students, and 67.9% and 72.8% for sixth‐year students. More importantly, 37% of residents‐in‐training believe the restorative procedure of choice for patients with an edentulous mandible is an implant supported by an overdenture while 43.9% believed a fixed implant supported prostheses as the preferred restorative procedure. Similarly, a majority of fifth (41.3%) and sixth year students (44.4%) selected fixed implant supported prostheses as their preferred procedure to treat edentulous mandibles. This was closely followed by conventional complete dentures which was selected by 38.4% and 36.3% of fifth‐ and sixth‐year students, respectively. In contrast, only 19.3% of residents selected the complete denture as their preferred option. There were statistically significant differences in the responses to questions on treating missing maxillary central incisors (*p* < 0.01) and edentulous mandible (*p* = 0.03) by student groups.

### 3.4 Learner's Perceptions on Implant Education and Future Practice

Overall, most respondents heard about implant treatment for the first time from their school lectures (64.4%), internet (32.3%), and clinical instructor (18.8%). Approximately, 80% of respondents reported topics on implants were discussed during predoctoral training. However, only 19.5% believe it was sufficiently covered. In addition, 56.5% rated their knowledge on implants as limited while only 5.7% rated their knowledge as proficient. Despite the dearth of implant education during predoctoral training, 96.3% of respondents believe implant education should be taught at the undergraduate level and the pedagogical method should at least incorporate theory, simulated practical, and clinical observation of a case (87.7%). Most respondents would like to incorporate implant treatment in their practice (89.3%) and would be interested in an online course on dental implant treatment (93.5%).

## 4 Discussion

This study is one of the first to comprehensively gather survey data from both faculty and learners across all dental schools in Nigeria, providing a representative overview of the current state of implant dentistry education in developing African countries. From the faculty's perspective, only about half of the dental schools required implant dentistry education. The three primary barriers to implementing a mandatory implant curriculum were a lack of curricular time, financial constraints, and a shortage of qualified faculty—challenges that align with findings from a global survey on implant education [22]. The same worldwide survey [22] indicated that in some developing countries, the lack of emphasis on implant dentistry at the predoctoral level contributed to the absence of an implant curriculum. Similar trends have been observed in certain European dental schools, where a lack of curricular time and the belief that implant dentistry should not be part of predoctoral education were also cited as barriers [23]. A 2017 survey of US dental schools found that all institutions had integrated dental implants into their predoctoral preclinical curricula, albeit with varying degrees of clinical exposure, primarily in restorative dentistry [24]. This emphasis on implant education within restorative departments parallels the findings of the present study. The growing momentum for modern implant education may have influenced faculty members' willingness to incorporate implant courses, particularly online programs. However, only one‐quarter of the dental schools surveyed had access to online implant courses. A survey from a dental school in Saudi Arabia revealed that while problem‐based learning was the most common teaching method (60%), e‐learning was also employed (15%) [25]. The faculty survey findings align with European consensus reports [26], other surveys [27], and global trends [28]. In the United States, there is also strong support for predoctoral implant training [29], with some dental schools even incorporating surgical training at the predoctoral level [30].

Regarding the learner survey, attitudes toward restoring a missing tooth with a single implant and treating complete edentulism with full‐arch implant‐supported prostheses improved with advancing education levels. This suggests that implant education influences treatment planning and the options that newly graduated practitioners offer their patients. Other studies have similarly indicated that predoctoral education impacts graduates' choices in implant practice [31]. A comprehensive predoctoral implant curriculum is crucial to ensuring that graduates are equipped to provide accurate diagnoses, consider implant options, develop treatment plans, and perform implant procedures for their future patients [31]. Our study, like previous research [32, 33, 34], suggests that education and clinical exposure shape students' attitudes and treatment decisions regarding implants. Continuing education appears to have a more significant impact at the graduate level than at the predoctoral level. However, across all levels, the learner survey demonstrated that confidence in implant proficiency remains limited. Therefore, methodical integration of laboratory and clinical experiences into both predoctoral and postgraduate curricula is necessary to ensure practice readiness [32, 33]. Both simulation and clinical experiences have been shown to enhance students' confidence and satisfaction with implant education [34]. Furthermore, all learners expressed a strong desire to expand their knowledge of implant dentistry, including both restorative and surgical aspects, through continuing education. This trend mirrors findings from a survey of Mongolian dentists, who also had limited exposure to implant education during dental school [35]. An overwhelming majority (> 90%) of learners expressed interest in incorporating dental implants into their future practices and indicated a preference for online continuing education programs, a trend that aligns with findings from the United Kingdom [35, 36]. As implant dentistry continues to expand, the demand for standardized and structured implant education has been increasingly recognized by clinicians, researchers, and educators worldwide [37].

This study has some limitations. First, while faculty surveys provided a comprehensive overview of implant education across all Nigerian dental schools, student participation was voluntary. As a result, only students interested in responding completed the survey. This convenience sampling may not fully represent the entire student body. Second, the faculty survey had a relatively low response rate (38 out of 55, approximately 69%), likely due to its voluntary nature rather than being mandatory. In addition, while we obtained responses from all dental schools in Nigeria, multiple faculty members were often responsible for implant dentistry education. When distributing multiple copies of the survey, not all faculty chose to respond. This may have introduced a bias toward course directors, as course faculty may have felt less compelled to participate. Third, the study did not include graduate dentists, who might have provided a clearer picture of general dental practice in Nigeria. Finally, the study did not account for the influence of social media and other available information that learners may have accessed, which could have influenced the results, particularly regarding the demand for online education.

It is the authors' belief that the inequities in implant education, that is being reported in Nigerian dental schools, will be greatly reduced if efforts are made to move forward with a plan for a unified curriculum that includes laboratory exercises. The educational intervention will ensure that graduates of these schools are exposed to evidenced‐based dental practice. It has the potential to be a great opportunity to share and transfer knowledge. Furthermore, it could serve to develop interest in the field of implantology. There is a window of opportunity to standardize the teaching of implant dentistry in Africa's populous nation. The use of online education resources will greatly enhance the transfer of knowledge to predoctoral students in Nigeria. Virtual education has become widely used in dental schools around the world due to the global pandemic. The internet and availability of online education channels has fundamentally changed mindsets about academic rigor. Open access journals, non‐peer reviewed journals, and online presentations by key opinion leaders representing corporate interests have become more accepted as legitimate sources of information. Based on the results of the faculty and learner surveys, the Kirkpatrick model [38] can be applied to enhance learners' competency in dental implant therapy. The model's four levels—reaction, learning, behavior, and results—can be systematically implemented, as outlined in Table 4. A 7‐year study implemented online learning for graduate students for implantology and periodontology had shown a promising success [39]. Securing industry support will be critical in overcoming the lack of financial resources that is being experienced presently. Decision should be made to allocate adequate curriculum to implant courses and to plan to train faculty to teach implant courses. Future studies should explore the impact of available continuing education programs in Nigeria, both in‐person and online, as well as the influence of social media on implant dentistry education. In addition, a longitudinal prospective study is needed to assess whether an integrated implant dentistry curriculum at the predoctoral and graduate levels affects patient care related to dental implants.

**TABLE 4:** An example of Kirkpatrick model for online dental implantology course.

Level of Assessment	Objective	Learning Methodology	Measure
Level 1: Reaction	Student's favorable feeling toward the online dental implant course	Self‐learning online lessons followed by in‐person seminars	Attitude toward dental implant therapy survey
Level 2: Learning	Student's knowledge and skills in dental implant therapy	Blended learning with online lesson together with faculty supervised hand‐on simulations	Pre‐ and post‐course evaluation using quizzes, written exams, and hand‐on exams
Level 3: Behavior	Student's implementation in the dental school's clinic	Supervise restorative and surgical implant therapy in patients	Entrustable Professional Activities (EPAs) for each implant procedures
Level 4: Results	Student's practices using implant therapy after graduation	Independent implantology practices	One‐year postgraduation survey of implantology practices

## 5 Conclusions

Dental implant education in Nigeria is primarily provided in the final year of predoctoral training and at the graduate level. However, only about half of the dental schools have integrated implant dentistry into their predoctoral curriculum, largely due to financial constraints, limited curricular time, and a lack of faculty expertise. Increased exposure to implant education in the final year of predoctoral training and at the graduate level appears to enhance confidence in offering implant treatment. There is a significant unmet need for online education and continuing education programs both during and after graduation to support learners who wish to incorporate implant dentistry into their future practice.
